# Downregulated Calcium-Binding Protein S100A16 and HSP27 in Placenta-Derived Multipotent Cells Induce Functional Astrocyte Differentiation

**DOI:** 10.1007/s12015-021-10319-3

**Published:** 2022-01-21

**Authors:** Yu-Che Cheng, Chi-Jung Huang, Wei-Chi Ku, Shu-Lin Guo, Lu-Tai Tien, Yih-Jing Lee, Chih-Cheng Chien

**Affiliations:** 1 Proteomics Laboratory, Department of Medical Research, Cathay General Hospital, Taipei, Taiwan; 2 Department of Biomedical Sciences and Engineering, National Central University, Jhongli, Taiwan; 3 School of Medicine, College of Medicine, Fu Jen Catholic University, New Taipei City, Taiwan; 4 Clinical Cancer Genetics, Department of Medical Research, Cathay General Hospital, Taipei, Taiwan; 5 Department of Biochemistry, National Defense Medical Center, Taipei, Taiwan; 6 Department of Anesthesiology, Cathay General Hospital, Taipei, Taiwan

**Keywords:** S100A16, HSP27, Astrocyte, PDMCs, Differentiation

## Abstract

Little is known about genes that induce stem cells differentiation into astrocytes. We previously described that heat shock protein 27 (HSP27) downregulation is directly related to neural differentiation under chemical induction in placenta-derived multipotent stem cells (PDMCs). Using this neural differentiation cell model, we cross-compared transcriptomic and proteomic data and selected 26 candidate genes with the same expression trends in both omics analyses. Those genes were further compared with a transcriptomic database derived from Alzheimer’s disease (AD). Eighteen out of 26 candidates showed opposite expression trends between our data and the AD database. The mRNA and protein expression levels of those candidates showed downregulation of HSP27, S100 calcium-binding protein A16 (S100A16) and two other genes in our neural differentiation cell model. Silencing these four genes with various combinations showed that co-silencing HSP27 and S100A16 has stronger effects than other combinations for astrocyte differentiation. The induced astrocyte showed typical astrocytic star-shape and developed with ramified, stringy and filamentous processes as well as differentiated endfoot structures. Also, some of them connected with each other and formed continuous network. Immunofluorescence quantification of various neural markers indicated that HSP27 and S100A16 downregulation mainly drive PDMCs differentiation into astrocytes. Immunofluorescence and confocal microscopic images showed the classical star-like shape morphology and co-expression of crucial astrocyte markers in induced astrocytes, while electrophysiology and Ca^2+^ influx examination further confirmed their functional characteristics. In conclusion, co-silencing of S100A16 and HSP27 without chemical induction leads to PDMCs differentiation into functional astrocytes.

**Graphical abstract:**

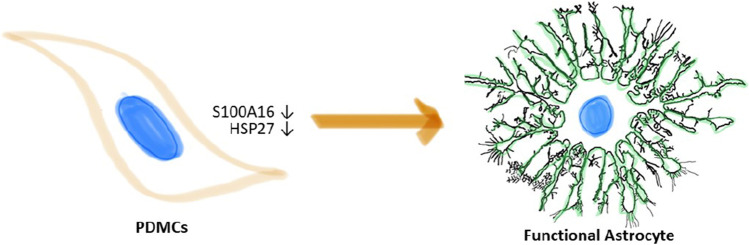

**Supplementary Information:**

The online version contains supplementary material available at 10.1007/s12015-021-10319-3.

## Introduction

Due to their properties of self-renewal and differentiation, stem cells, including mesenchymal stem cells and induced pluripotent stem cells (iPSCs), are promising for regenerative medicine [1]. Previously, our group isolated stem cells from human placenta, termed placenta-derived multipotent stem cells (PDMCs) and found that they can be induced and differentiated into hepatocytes, bone cells and neurons [2, 3]. We also found that, under chemical induction, heat shock protein 27 (HSP27) downregulation leads to a highly efficient PDMC differentiation into glutamatergic neurons [4]. Despite iPSCs’ potential in biomedical research and personalized regenerative medicine, however, there remain several challenges such as genomic instability [5], the epigenetic memory from the somatic cell source during reprogramming [6] and the altered iPSC characteristics and differentiation potentials by expression of reprogramming factors [7]. Thus, its availability and naïve background make PDMCs a good resource for the study and application of regenerative medicine.

Unwanted proliferation or differentiation has restricted the clinical application of iPSCs [8], as the tumorigenicity of these cells is long recognized [9]. Understanding the mechanisms that control the differentiation and proliferation of implanted stem cells is crucial for future implantation of the tissues or organs derived from these cells. To better understand the underlying mechanisms, we applied a double-cross-comparison screening strategy to match the most consistently expressed molecules in genomic and proteomic databases with the genes found expressed in clinical tissue.

Astrocytes, the most abundant cells in the CNS, play critical roles in the maintenance of neural homeostasis; they are involved in neurotransmitter trafficking and recycling [10], clearance of neuronal waste [11], and protection against oxidative stress [12]. Astrocyte dysfunction has been found to be involved in some neurological disorders, such as sporadic amyotrophic lateral sclerosis epilepsy, autism, lysosomal storage diseases, and Alzheimer’s disease (AD) [13, 14]. Despite the effectiveness of some methods for the differentiation of embryonic stem cells [15] and human iPSCs [16] into astrocytes, either the differentiation efficiency was low or the process duration long—on the order of months—which limits their clinical applications. In addition, in most cases, this differentiation was induced by defined chemical environments [17, 18]. For example, overexpression of the transcription factors SOX9 and NFIB in human pluripotent stem cells can induce astrocyte differentiation [19, 20]. In contrast, herein we provide another approach involving co-silencing of HSP27 and S100A16, selected through a double-cross-comparison screening strategy, to efficiently induce PDMCs differentiation into functional astrocytes within three weeks, thus largely reducing the time for astrocyte differentiation.

## Materials and Methods

### PDMC Cell Culture

PDMCs were obtained as previously, with some modifications [21]. Healthy donors provided fully informed consent, and the study protocol was approved by the Institutional Review Board of Cathay General Hospital under approval number CGH-P105098. After arriving in the laboratory, placentas were cut into pieces using sterilized scissors and washed with phosphate-buffered saline. Next, adequate volumes of normal saline with 0.25% trypsin–EDTA (Gibco/Life Technologies, Carlsbad, CA, USA) were poured over the tissue samples, which were then incubated for 10 min at 37 °C. Then, the samples were centrifuged, and the cells resuspended in DMEM (Gibco/Life Technologies, Carlsbad, CA, USA) with 10% FBS (HyClone/GE Healthcare, Novato, CA, USA), 100 U/mL penicillin and 100 g/mL streptomycin (MilliporeSigma, St. Louis, MO, USA). Cell cultures were maintained at 37 °C with 5% CO_2_.

### Neural Differentiation

For chemical induction of PDMC neural differentiation, cells were cultured in complete DMEM with 0.4 mM 3-Isobutyl-1-methylxanthine (IBMX) without FBS, as previously reported [4]. For gene silencing-mediated induction of neural differentiation, HSP27 and S100A16 were co-silenced with shRNAs as described below.

### Microarray and Cross-Comparison of Microarray and Proteomics Data

To investigate the differentially expressed genes in response to IBMX treatment, microarray hybridization (Agilent SurePrint G3 Human GE 8 × 60 K oligonucleotide microarrays; Agilent Technologies, USA) was performed as described in our previous report [22]. All raw microarray data was uploaded to Gene Expression Ominibus (GEO) / NCBI (accession number: GSE139656). Briefly, the log ratio was defined as log2 (Y/N), where Y was the gene expression level in PDMCs after 12 h or 24 h of IBMX treatment and N the gene expression level in PDMCs without IBMX treatment (0 h). Genes with log2 ratios > 1 or <  − 1 were defined as significantly differentially expressed. In addition, cell lysates from PDMCs with or without IBMX treatment at different times (12 h, 24 h, and 48 h) were subjected to proteomics analysis. We used the standard deviation (SD) to evaluate protein level differences in response to IBMX treatment [23]. The proteins in the highest 10% (SD > 1.28) and lowest 10% (SD <  − 1.28) expression in IBMX-treated PDMCs were plotted and assessed relative to those from cells without IBMX treatment. Candidates with similar significant expression patterns in microarray hybridization and proteomics analysis were subjected to further validation.

### Imaging Cytometry Analysis

Cells were first fixed on NucleoCounter NC-3000 Image Cytometer slides (ChemoMetec, Gydevang, Denmark) and individually stained with the following antibodies, including rabbit anti-Microtubule-Associated Protein 2 (MAP2, #AB5622, Chemicon/ Merck Millipore), mouse anti-Tubulin beta III isoform (Tuj1, #MAB1637, Chemicon/ Merck Millipore), mouse anti-plasma membrane calcium ATPase (PMCA ATPase, #MA1-34528, Thermo Scientific), rabbit anti-Vesicular glutamate transporter 1 (VGluT1, #ab72311, Abcam), rabbit Anti- N-Methyl D-Aspartate Receptor Subtype 2B (NMDAR2B, #ab65783, Abcam), rabbit anti-Synaptosomal-Associated Protein 25 (SNAP25, #ab109105, Abcam), mouse anti-Glial Fibrillary Acidic Protein (GFAP, #MAB3402, Chemicon/ Merck Millipore), mouse anti-Aldehyde Dehydrogenase 1 Family Member L1 (ALDH1L1, #ab56777, Abcam), rabbit anti-Glutamine Synthetase (GS, #SC-6640, Santa Cruz), mouse Anti-Glutamate Decarboxylase 2 (GAD65, #ab26113, Abcam), rabbit anti-Choline Acetyltransferase (ChAT, #ab68779, Abcam), and rabbit anti-Tyrosine Hydroxylase (TH, #ARG52461, Arigobio). The FlexiCyte™ imager mode was used to obtain digitalized data. All stain intensities were automatically read and charted.

### HSP27 and/or S100A16 Silencing in PDMCs

The procedure was performed according to our previous publication, with slight modifications [4]. pLKO.1-puro plasmid-based short hairpin RNAs (shRNAs), including shLuc (luciferase shRNA), shHSP27 (HSP27 shRNA) and shS100A16 (S100A16 shRNA), were acquired from the National RNAi Core Facility (Academia Sinica, Taiwan) and used for silencing of luciferase, HSP27 and S100A16, respectively, in PDMCs. The purified shRNA plasmids, including shLuc, shHSP27, and shS100A16, were individually transfected into H293T cells using Lipofectamine (Invitrogen/Life Technologies, CA, USA). The cells were also transfected with an envelope expression plasmid (pMD2.G) and a packaging vector (psPAX2) to generate shRNA-containing lentiviruses. The produced lentiviruses were then used to infect PDMCs according to a protocol by the National RNAi Core Facility (Academia Sinica, Taiwan). PDMCs with stable shRNA expression were selected using puromycin (0.5 μg/mL; Calbiochem/Merck Millipore, CA, USA).

### Calcium Influx

The induced astrocyte-like cells and control cells were first cultured in a 96-well microplate and incubated with 2 μM Calbryte 520 calcium dye (#20651, AAT Bioquest, CA, USA) in phenol red-free DMEM (#30153028, Gibco/Thermo Fisher) for 1 h at 37 °C. The Calbryte 520 calcium dye-containing medium was replaced by low-K^+^ Tyrode’s solution (2 mM CaCl_2_, 140 mM NaCl, 1 mM MgCl_2_, 4 mM KCl, 10 mM glucose, and 10 mM HEPES, pH 7.2), the cells were incubated for another 30 min, and the absorbance read with an excitation/emission wavelength of 490/525 nm at room temperature with a Synergy HT Multi-Mode microplate reader (BioTek, VT, USA) to obtain the baseline. After a 90 s baseline recording, 3.5 U/ml human thrombin in low-K^+^ Tyrode’s solution was added, and the experiments run at an excitation/emission wavelength of 490/525 nm at room temperature. The results were normalized by the initial baseline values and plotted.

### Electrophysiology

Ten days after co-silencing of S100A16 and/or HSP27, cells were plated onto 12 mm coverslips at a 1 × 10^4^ density. After 2–3 days, electrophysiological recordings were performed. Membrane currents were recorded using whole-cell recordings by patch-clamp. Patch pipettes were prepared in-house from glass capillaries (Kimble). The capillaries were pulled and fire-polished in order to obtain a tip resistance of 3–4 MΩ with solution. Data were recorded at 10 kHz (Axon MultiClamp 700B amplifier with Pulse software; Signal 4.08). The series resistance was maintained < 10 MΩ. Recordings with leak currents > 100 pA were discarded. The protocol of voltage stimulation from the holding potential (Vh) of − 60 mV, stepped the membrane potential to − 100 mV for 200 ms before a 100 ms long depolarizing ramp to + 120 mV. In voltage-step measurements, the Vh was − 60 mV, with steps from − 100 mV to + 120 mV in 20 mV increments over 200 ms. Ten seconds was used as the interval between pulses in both protocols. After establishing the whole-cell recording configuration, the resting membrane potential was obtained for 120 s using the amplifier analog circuit. Electrophysiological experiments were performed by perfusing the clamped cell with a standard bath saline solution (119 mM NaCl, 2.5 mM KCl, 2.5 mM CaCl_2_, 26.2 mM NaHCO_3_, 1 mM NaH_2_PO_4_, 1.3 mM MgSO_4_, 11 mM glucose, pH 7.4), and osmolality adjusted by mannitol. The intracellular pipette solution was prepared as follows: 145 mM KCl, 1 mM MgCl_2_, 2 mM EGTA, 0.2 mM CaCl_2_, 2 mM Mg-ATP, 0.3 mM Na_3_-GTP, 10 mM HEPES (pH 7.2), and osmolality of 300 ~ 310 mOsm kg^−1^ by mannitol. A gravity-driven microperfusion system with a 2 ml/min flow rate was used to apply the extracellular solution.

### Statistical Analysis

Data were analyzed using SPSS, version 21.0 (IBM, New York, NY, USA). Quantitative variables are described in means ± SD. One-way ANOVA was used to compare the differences between groups followed by Bonferroni post hoc test. All tests were two-sided. For pair comparison, the Student’s T test were used. *P* < 0.05 considered statistically significant.

Shotgun proteomics methods including protein digestion, dimethyl labeling, strong cation exchange separation, and MS data processing are listed in the Supplements. Immunoblotting procedures including antibodies employed, total RNA extraction and quantitative real-time PCR method, immunofluorescence, and Laser scanning confocal fluorescence microscopy procedures and used antibodies are listed in the Supplements S1.

## Results

### Double-Cross-Comparison Screening Strategy for Identification of Crucial Genes in Neural Differentiation

**Fig. 1:**
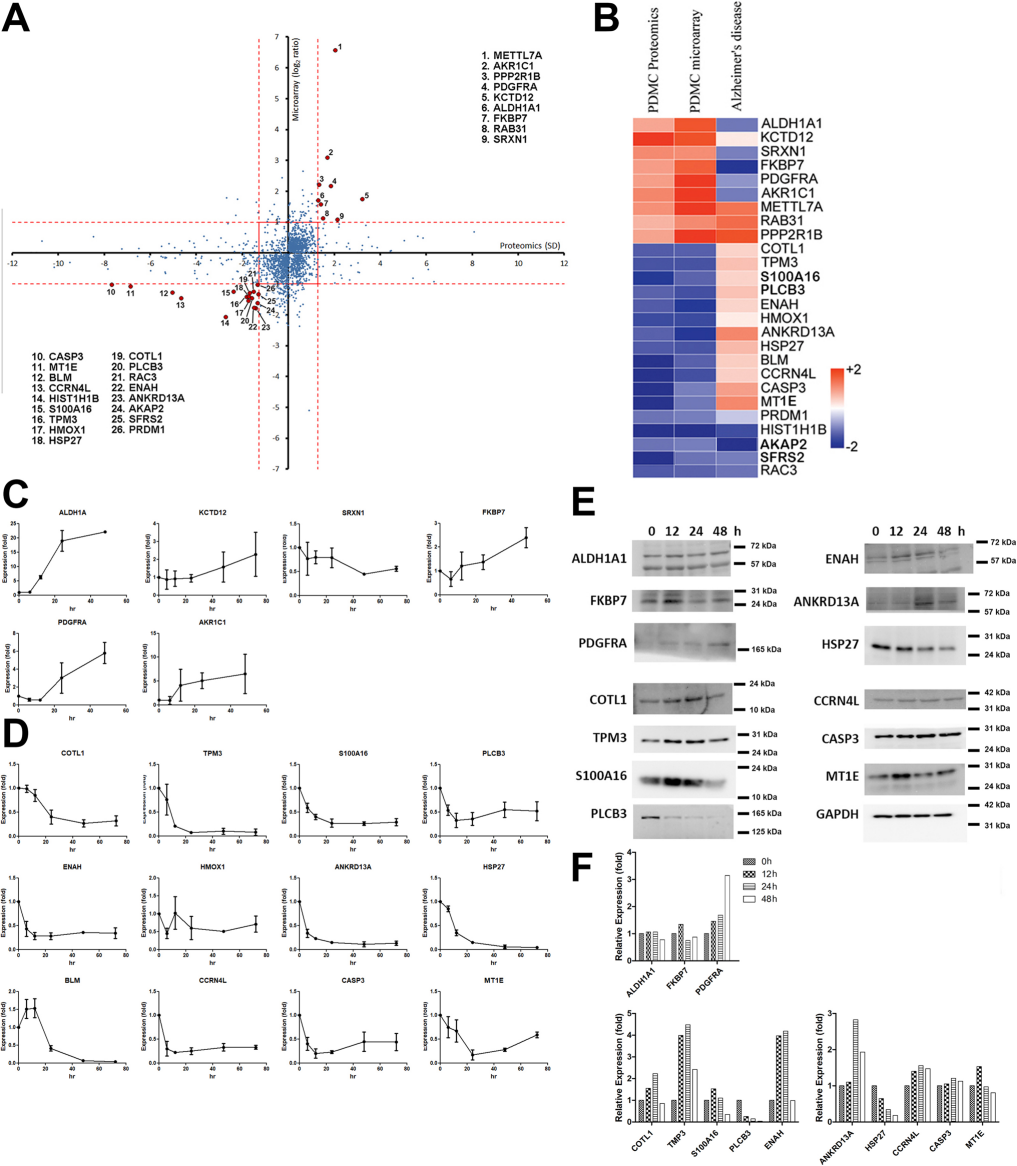
Double-cross-comparison for screening neural regeneration-related genes. (**A**) PDMCs were induced to differentiate into neural cells by 0.4 mM IBMX. mRNAs and proteins were extracted and used for mRNA expression microarray and shotgun proteomic analyses, respectively. The results from the two high-throughput omics approaches were compared and plotted (blue dots). In order to investigate the crucial genes with the same expression trends, we set the exclusion criteria as 1.28 in log_2_ notation. Using this strategy, we narrowed the list of gene candidates to nine upregulated genes and 17 downregulated genes at both the mRNA and protein levels. The selected genes are showed as red dots and indicated with names. (**B**) To address Alzheimer’s disease (AD)-relevant genes, we compared the expression of these 26 genes with expression array data originating from an AD patient. In this analysis, we double comparison of genes with opposite expression patterns because AD is a neurodegenerative disease. (**C** and **D**) mRNA expression of the selected genes from the double-cross-comparison strategy verified by qPCR in the PDMCs induced neural cell model (**C** for upregulation and **D** for downregulation). We removed KCTD12, SRXN1 and AKR1C1 from the upregulated candidate genes in C, and HMOX1 and BLM from the downregulated candidate genes in D because their mRNA expression showed opposite regulation patterns or because their expression showed too much variation. (**E**) The remaining genes were further tested for protein expressions by immunoblotting. From those results, PDGFRA, S100A16, PLCB3, HSP27, and MT1E showed the same trends for protein expression as for mRNA expression; therefore, we kept these genes in the candidate list. The other proteins showed opposite regulation patterns or exhibited no changes in protein expression during differentiation. (**F**) The band intensities were digitized from the immunoblotting results from (**E**). The results of each proteins were all divided by the intensity of GAPDH of individual time points to show the relative expression fold. Abbreviations used: methyltransferase Like 7A, METTL7A; aldo–keto reductase family 1 member C1, AKR1C1; serine/threonine-protein phosphatase 2A 65 kDa regulatory subunit A beta isoform, PPP2R1B; platelet-derived growth factor receptor A, PDGFRA; BTB/POZ domain-containing protein KCTD12, KCTD12; aldehyde dehydrogenase 1 family, ALDH1A1; FK506 binding protein 7, FKBP7; ras-related protein rab-31, RAB31; sulfiredoxin 1, SRXN1; caspase 3, CASP3; metallothionein-IE, MT-IE; bloom syndrome protein, BLM; nocturnin, CCRN4L; histone H1.5, HIST1H1B; S100 calcium-binding protein A16, S100A16; tropomyosin alpha-3 chain, TPM3; heme oxygenase (decycling) 1, HMOX1; heat shock protein 27, HSP27; coactosin-like protein,COTL1; 1-phosphatidylinositol-4,5-bisphosphate phosphodiesterase beta-3, PLCB3; ras-related C3 botulinum toxin substrate 3, RAC3; protein enabled homolog, ENAH; ankyrin repeat domain-containing protein 13A, ANKRD13A; A-kinase anchor protein 2, AKAP2; splicing factor, arginine/serine-rich 2, SFRS2; PR domain zinc finger protein 1, PRDM1

We used neural differentiation model derived from PDMCs as cell model [4]. The mRNAs and proteins were collected respectively from the differentiated cells. The mRNAs were used for probing of a human oligonucleotide microarray. The proteins were digested with trypsin and then labeled with dimethyl groups for a shotgun proteomic approach. The microarray results were cross-compared with the shotgun proteomic data at the expression level, and the results plotted in Fig. 1A. We considered that the crucial genes in neural differentiation, our candidate genes, would be those with the same expression trends at transcriptional and translational levels. For the results in Fig. 1A, we set the exclusion criteria as 1.28 in log_2_ notation. We found 26 gene candidates fitting our criteria (red dots in Fig. 1A). Later, to screen neural regeneration-related genes, we cross-compared these 26 candidate genes with a publicly available AD microarray database. The goal of the second cross-comparison was to search candidate genes with opposite expression patterns between the regeneration and degeneration databases. We removed the METTL7A, RAB31, PPP2R1B, PRDM1, HIST1H1B, AKAP2, SFRS2 and RAC3 genes from our candidate list because the expression trends of these genes in AD were the same as in our two types of omics data (Fig. 1B, all red or all blue boxes in the three databases). We performed qPCR to verify the expression of the remaining 18 candidate genes in our list (Fig. 1C, upregulated genes and 1D, downregulated genes). We found that the mRNA expression levels of KCTD12, SRXN1, AKR1C1, HMOX1 and BLM were not in accordance with our transcriptomic results and thus were removed from our candidate list. Next, we immunoblotted the remaining 13 candidate genes (Fig. 1E), and the band intensities were quantified. All intensities of each band were normalized with GAPDH expressions of individual time point (Fig. 1F). The results showed that in the upregulation group, only platelet-derived growth factor receptor alpha (PDGFRA) fit our expectations and it may have some effects of promoting differentiation. In the downregulation group, HSP27 1-phosphatidylinositol 4,5-bisphosphate phosphodiesterase beta-3 (PLCB3) were expressed as expected. As to the S100A16 and metallothionein-1E (MT1E), except the expression at 0 h, all other time points showed gradually down-regulated manner.

### HSP27 and S100A16 Downregulation is Crucial for Neural Differentiation

**Fig. 2:**
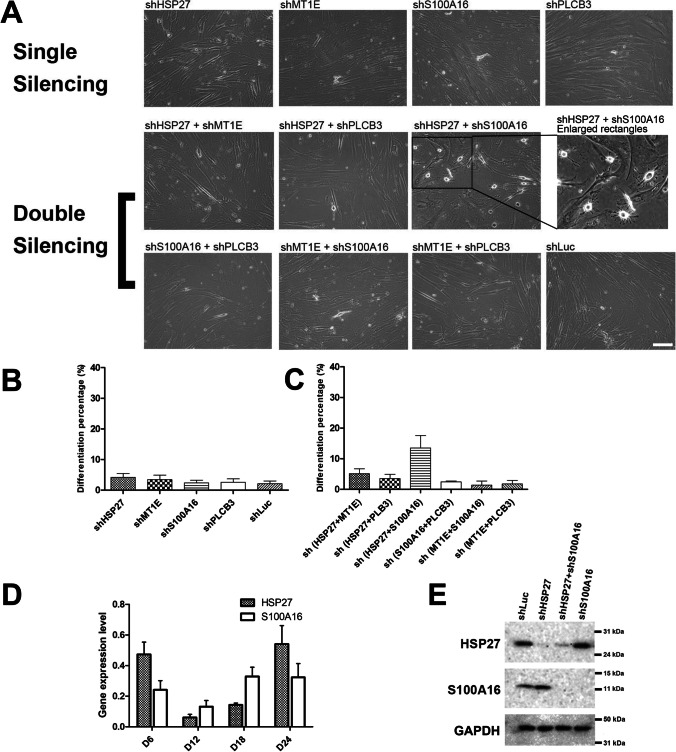
Cell morphology changes under various gene manipulation combinations in PDMCs. (**A**) Phase contrast images of cells under silencing of various gene combinations. PDMCs were infected with viruses containing shRNAs specific to HSP27 (shHSP27), MT1E (shMT1E), S100A16 (shS100A16) and PLCB3 (shPLCB3). Cells infected with viruses containing Luciferase shRNA (shLuc) were used as infection controls. We performed single gene silencing (upper row) and double gene silencing (middle and lower rows). The cell images were taken 12 days after virus infection. The cells with HSP27 and S100A16 double silencing showed the highest neural cell differentiation among the groups (middle row, shHSP27 + shS100A16). For better characterization of induced neural cells, an enlarged image is shown in the very right panel of middle row. The relative position of the enlarged rectangle is indicated by a black frame in the original image. Scale bar: 100 μm. (**B** and **C**) Percentage of astrocyte-like cells under various combination of gene silencing. The phase contrast images of PDMCs with single silence or various double silence combination were taken 12 days after infection. Six images were randomly selected of each experimental groups and the astrocyte-like cells were counted according to their morphology. (**D** and **E**) Determination of HSP27 and S100A16 mRNA and protein expression level. PDMCs were co-infected with viruses containing shRNAs specific to HSP27 (shHSP27) and S100A16 (shS100A16). Cells infected with viruses containing shRNA specific for silencing of luciferase (shLuc) were used as controls. After infection, cells were incubated for 12 (D12), 18 (D18) or 24 days (D24). Cells were harvested and mRNA extracted for qRT-PCR at those time points. The proteins in the cells at D18 were used for Western blotting

**Fig. 3:**
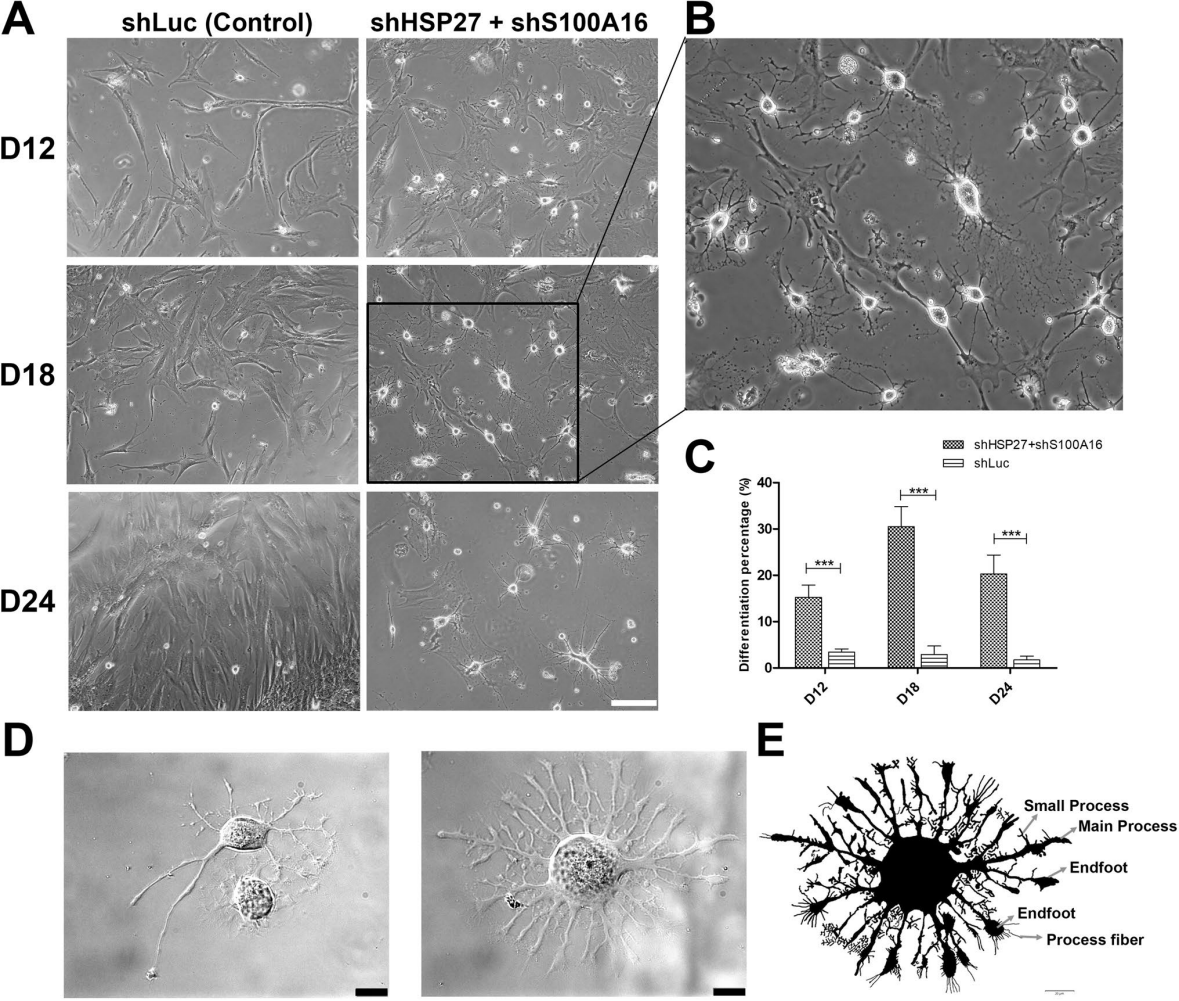
PDMCs morphology with HSP27 and S100A16 silencing at different time points. (**A** and **B**) Phase contrast images of cells with HSP27 and S100A16 double silencing. Compare to the cells silenced with Luciferase (shLuc Control, left column), there were nearly no induced astrocyte. The induced cells showed many dendritic processes at D12 and reached their optimum at D18 without chemical inducers (shHSP27 + shS100A16, right column). For better characterization of induced astrocyte, a part of image at D18 were selected and enlarged to observe the formed astroglial network. The relative position of the enlarged rectangle is indicated by a black frame in the original image. Scale bar: 100 μm. (**C**) Percentage of induced astrocytes with HSP27 and S100A16 co-silencing. The bar chart shows the percentage of induced astrocyte quantified from the above conditions. The induced astrocytes were counted according to their morphology. * * *: *p* < 0.001. (**D** and **E**) High resolution images of induced astrocytes. Induced astrocytes (D18) showed a typical star-shaped morphology. The descriptive image originated from the middle image was processed by Photoshop and the specific fine structure of filamentous processes were indicated by arrows. The images were taken using Olympus BX51W Scientifica system coupled with DAGE-MTI IR-1000 CCD. Scale bar: 20 μm

To evaluate the physiological roles played by these four downregulated genes, we silenced these genes alone or in various double combinations via lentivirus-mediated silencing. By observing cell morphology, we found that silencing of each of the four candidate genes individually showed little effect (Fig. 2A, row 1, shHSP27, shMT1E, shS100A16 and shPLCB3), as well as for luciferase silencing (used as a control; Fig. 2A, row 3, at the very right panel, shLuc). However, in the double silencing experiments, several combinations elicited neural-like morphology, especially in the shHSP27 plus shS100A16-treated group (Fig. 2A, row 2, middle panel, shHSP27 + shS100A16). The induced cells could be better identified with enlarged rectangle image (Fig. 2A, row 2, right panel, shHSP27 + shS100A16 Enlarged rectangle). The percentage of astrocyte-like cells out of total cells compared to all other gene silence groups were quantified (Fig. 2B for single gene silence; Fig. 2C for double gene silence). The mRNA and protein expression levels of HSP27 and S100A16 were examined from the cells with shHSP27 plus shS100A16-treatment and the results confirmed the downregulated HSP27 and S100A16 in the cells (Fig. 2D for mRNA and 2E for protein expression levels). Some of the induced cell morphology by silencing HSP27 and S100A16 showed star-shaped cells and those cells were resemble as astrocyte indicating that downregulation of these two genes might lead stem cells differentiated into astrocyte. It is worth to notice that there were no chemical inducers, such as IBMX, in these experiments; only the HSP27 and S100A16 genes were silenced. We also conducted the combination of 4-gene silence and the various combination of 3-gene silence; the results were presented in the Supplement S2. However, neither one of them showed better differentiation ability than co-silencing of HSP27 and S100A16. We therefore applied co-silencing of HSP27 and S100A16 in PDMCs and observed cell morphology at 12, 18, and 24 days after cells infected with lentiviruses containing shHSP27 and shS100A16. We found that many differentiated cells showed star shapes, a typical astrocytic shape and developed with ramified, stringy and filamentous processes (Fig. 3A). Also, some of them connected with and formed continuous network (Fig. 3A, D18, enlarged rectangle). For each experimental condition, the induced astrocytes were normalized and quantified (Fig. 3C). According to cell images and quantification results, 18 days was the optimal time point for astrocytic differentiation. Thereafter, we captured images of the induced cells using higher resolution microscopy with IR CCD and the cells showed a typical stellate morphology (Fig. 3D). We also draw descriptive figure base on IR image (Fig. 3E). From descriptive image, we found the induced astrocyte contained astrocytic main and small processes, well differentiated endfoot structures and we also notice that there were some process fibers extended from a particular endfeet, probably because they want to explore and touch neighboring neuron or endothelial cell. All these evidences led us to consider the differentiated cells are astrocytes.

### Characterization of Differentiated Cell Types

We next tried to characterize the types of differentiated cells derived from HSP27 and S100A16 co-silencing. We used the optimum conditions for astrocyte differentiation mentioned above that is 18 days post co-silencing and stained the differentiated astrocytes with various neural and glial markers. From the fluorescence quantification results shown in Table 1, we found that silencing of either HSP27 or S100A16 alone exerted little effect on astrocyte or neuron differentiation markers, except for neuron-specific class III β-tubulin (TUJ1) with 13.88% positive rate in shHSP27-treated cells and glial fibrillary acidic protein (GFAP) with 6.89% positive rate in shS100A16-treated cells. However, in case of double silencing, we found that shHSP27 and shS100A16 together significantly enhanced astrocytic markers expressions such as microtubule-associated protein 2 (MAP2) with 7.12% positive rate, vesicular glutamate transporter 1 (vGLUT1) with 17.28% positive rate, GFAP with 24.94% positive rate, glutamine synthetase (GS) with 9.57% positive rate, and aldehyde dehydrogenase 1 family member L1 (ALDH1L1) with 13.19% positive rate. Compare to control cells, there were nearly no expression of the astrocytic markers mentioned above. Other markers specific to cholinergic neurons (choline acetyltransferase, ChAT), dopaminergic neurons (tyrosine hydroxylase, TH), GABAergic neurons (glutamate decarboxylase 65, GAD65) and glutamatergic neurons (N-methyl-d-aspartate receptor 2B, NMDAR2B and synaptosomal-associated protein of 25 kDa, SNAP25) did not exhibit significant expression changes. The results indicate that silencing of HSP27 and S100A16 leads to PDMC differentiation into astrocytes.

**Table 1:** Fluorescence quantification of induced neural cell by various neuron and astroglial markers. PDMCs with HSP27 and S100A16 co-silencing 18 days post co-silencing were probed with various neuron and astroglial marker antibodies, and appropriate secondary antibodies with FITC conjugation were applied thereafter. DAPI was used to stain the cell nucleus. Cells with immunofluorescence signals were subjected to fluorescence quantification in an NC 3000 image cytometer. The percentage of each fluorescence-positive cells over DAPI-positive cells were calculated. Data were collected from three independent experiments. The mean values of each marker in each experimental group were compared to the mean values in the shLuc group for statistical calculation. * *p* < 0.05; ** *p* < 0.01; *** *p* < 0.001. Abbreviations used: Microtubule-Associated Protein 2, MAP2; Neuron-specific Class III β-tubulin, TUJ1; ATPase, calcium pump of the plasma membrane ATPase; vesicular glutamate transporter 1, vGLUT1; N-Methyl-d-Aspartate Receptor 2B, NMDAR2B; synaptosomal-associated protein of 25 kDa, SNAP25; glial fibrillary acidic protein, GFAP; aldehyde dehydrogenase 1 family member L1, ALDH1L1; Glutamine Synthetase, GS; Glutamate decarboxylase 65, GAD65; Choline acetyltransferase, ChAT; Tyrosine hydroxylase, TH

	shLuc		shHSP27 + shS100A16		shHSP27		shS100A16	
Markers	Mean (%)	SD (%)	Mean (%)	SD (%)	Mean (%)	SD (%)	Mean (%)	SD (%)
MAP2	0.84	0.22	7.12*	1.56	3.11	0.30	1.84	0.07
TUJ1	0.92	0.02	2.19	0.86	13.88**	2.55	1.35	0.01
ATPase	1.04	0.74	0.42	0.20	0.15*	0.04	0.48	0.06
vGLUT1	0.83	0.06	17.28**	1.54	0.57	0.16	9.69	3.61
NMDAR 2B	1.03	00.1	0.39	0.06	0.45	0.23	0.35	0.36
SNAP25	0.70	0.04	0.74	0.48	0.21	0.04	0.43	0.12
GFAP	0.68	0.27	24.94***	1.29	0.88	0.16	6.89*	2.02
ALDH1L1	0.80	0.32	13.19***	1.64	4.96	0.50	1.21	0.07
GS	0.82	0.26	9.57*	0.66	2.94	0.71	4.52	1.18
GAD65	1.07	0.76	0.16	0.54	0.35	0.28	2.93	1.90
ChAT	1.01	0.01	3.68	1.20	2.64	1.12	0.22	0.03
TH	0.88	0.13	1.07	0.01	0.72	0.01	1.06	0.09

## Confirmation of Astroglial Differentiation with Astrocyte Markers

**Fig. 4:**
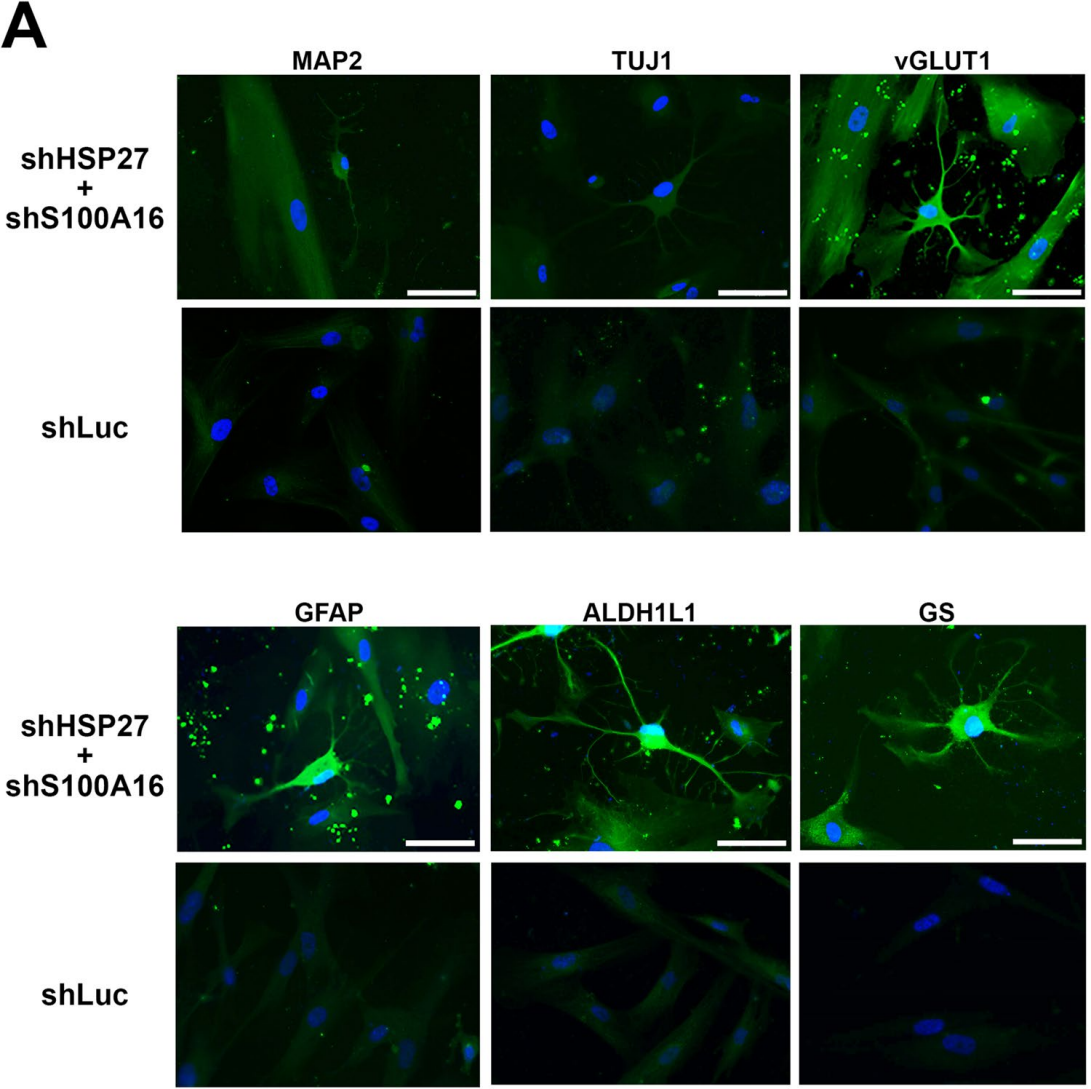

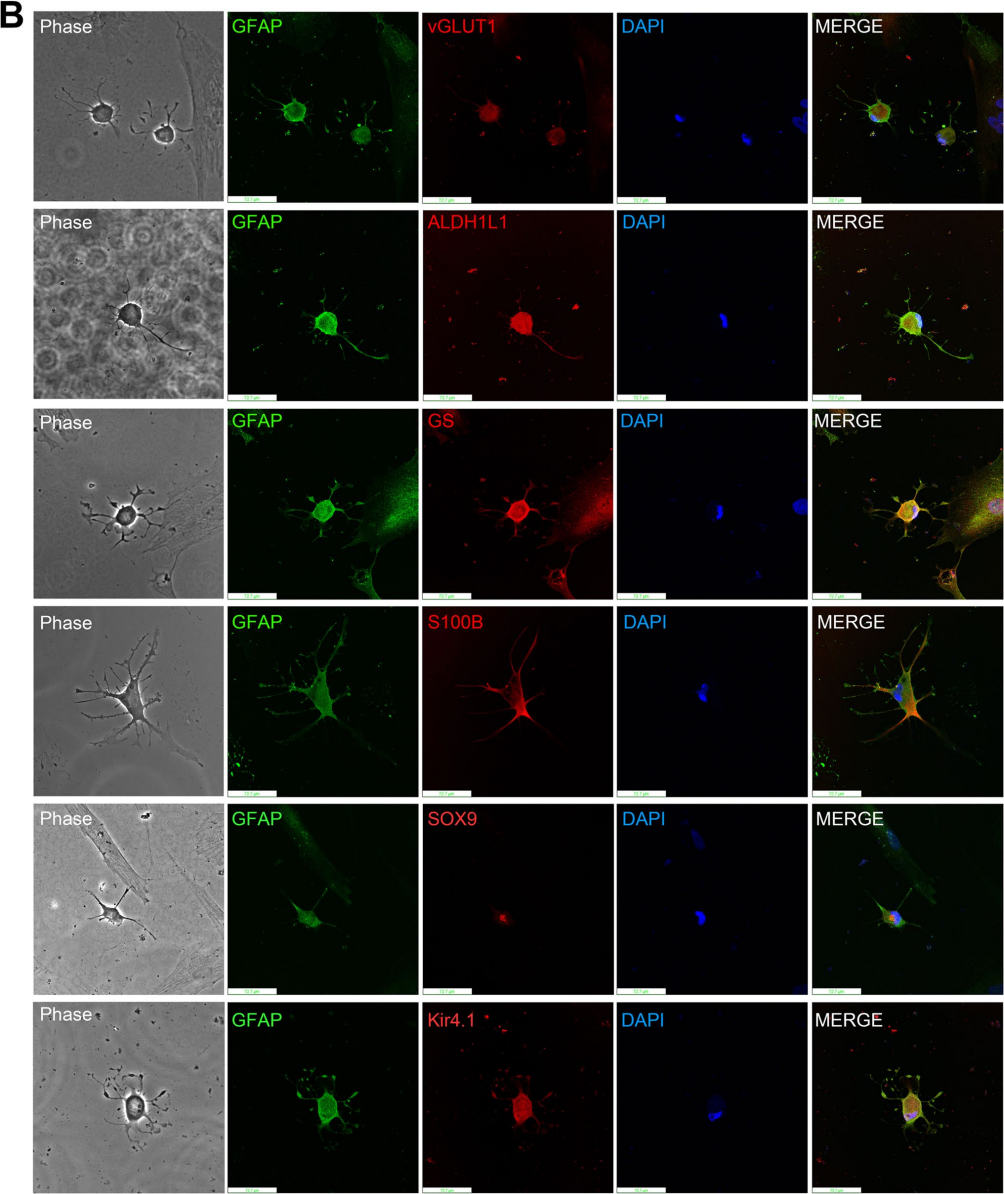
Co-silencing of HSP27 and S100A16 directs PDMCs differentiation into astrocytes. (**A**) Immunofluorescence imaging of induced astrocytes. PDMCs with co-silencing of HSP27 and S100A16 (shHSP27 + shS100A16) 18 days after virus infection. The induced astrocytes were probed with primary antibodies specific against MAP2, TUJ1, vGLUT1, GFAP, ALDH1L1, and GS following appropriate FITC-conjugated secondary antibody. PDMCs with silencing of luciferase (shLuc) were also stained with specific antibodies to demonstrate the specificity of each immunostaining. Cell nuclei were stained with DAPI. Scale bar: 50 μm. (**B**) Laser scanning confocal fluorescence microscopy images of induced astrocytes. PDMCs with co-silencing of HSP27 and S100A16 to induce astrocyte differentiation double stained with anti-GFAP antibody (Green) combined with other markers (Red) including vGLUT1, ALDH1L1, GS, S100B, SOX9 and KIR4.1. Scale bar: 72.7 μm

We next performed immunofluorescence staining of induced astrocyte (D18) to confirm cell lineage with astrocytic markers including MAP2, TUJ1, vGLUT1, GFAP, ALDH1L1, and GS (Fig. 4A). The results, in accordance with previous immunofluorescence intensity counts, showed slightly faint MAP2 and TUJ1 immunofluorescence but intense vGLUT1, GFAP, ALDH1L1, and GS immunofluorescence. The results demonstrated that not only the differentiated cells could be stained for these astrocyte markers but also exhibit a typical astrocytic morphology with their processes arranged in filamentous star-shaped patterns. Next, to ensure the co-expression of astroglial specific markers, we performed double staining in induced astrocytes with GFAP and other astroglial markers. The images were captured using laser scanning confocal fluorescence microscopy (Fig. 4B). GFAP staining served to locate induced astrocytes and to better observe other markers. The induced cells showed the typical star-shaped astrocyte morphology (Fig. 4B, Phase column and GFAP column, Green), with some astrocytic end-feet and 4 to 6 main processes in phase contrast images. Meanwhile, positive astrocytic markers including vGLUT1, ALDH1L1, GS, S100 calcium-binding protein B (S100B) (Fig. 4B, middle column, Red) demonstrate the astrocytic nature of differentiated cells. An astrocyte-specific nuclear marker, SOX9, showed a dense staining in the nucleus which in agreement with previous reports [24]. We also performed the immunostaining of an inwardly rectifying potassium channel, KIR4.1 (Fig. 4B, the lowest images in the middle column, Red), which showed highly expressed astrocyte. We observed that KIR4.1 were expressed in the cell membrane which demonstrated the fact that KIR4.1 is a potassium channel [25]. We also observed the KIR4.1 also enriched expressed in the endfeet of induced astrocyte, this fact is in accordance with other report [26]. All these results indicated the PDMCs was differentiated into astrocyte.

### Functional Characterization of Induced Astrocytes

**Fig. 5:**
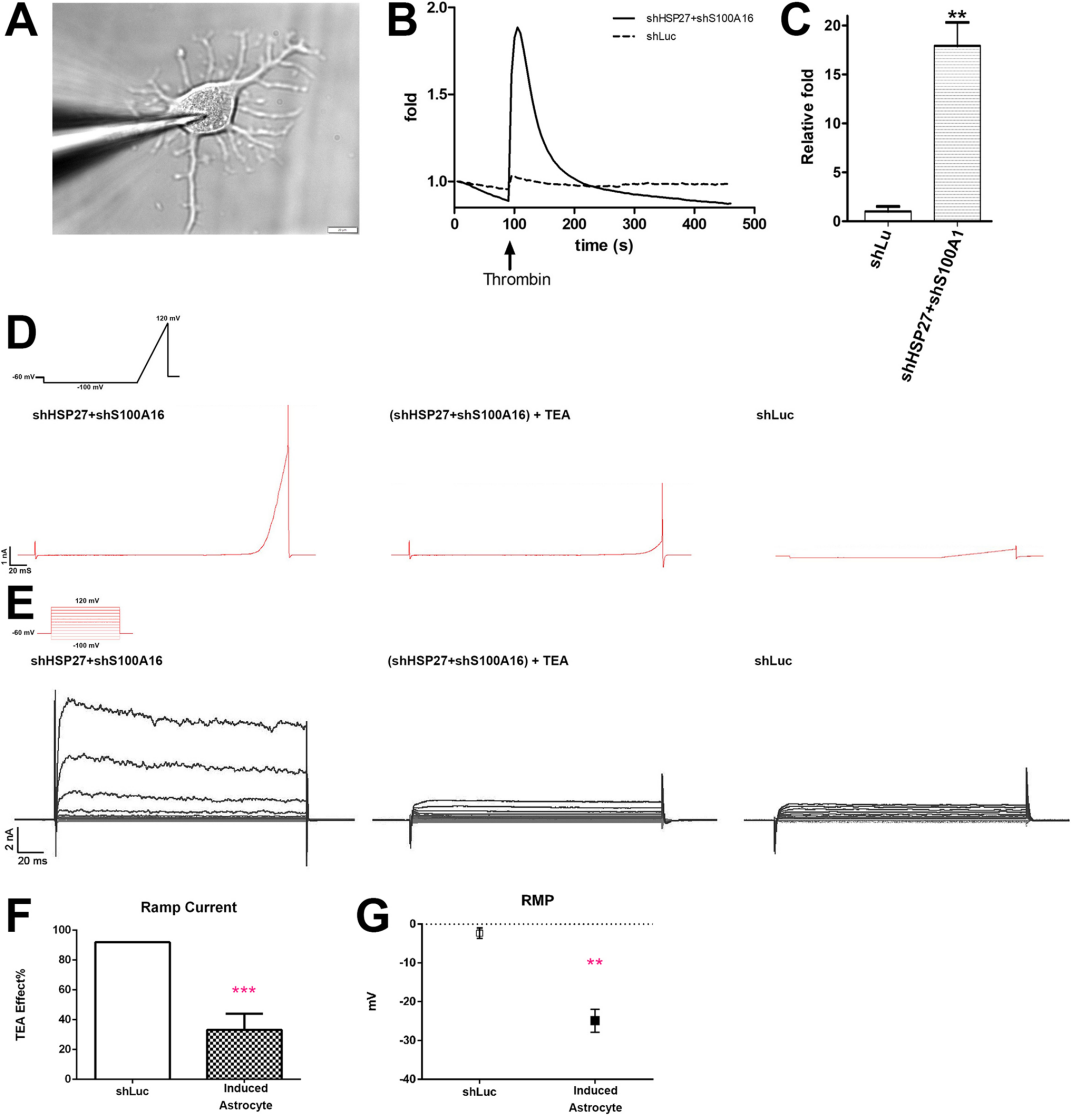
Electrophysiological recording of induced astrocytes derived from co-silencing of HSP27 and S100A16. (**A**) The induced astrocyte (D18) attached with the recording micropipette. The image was captured by Olympus BX51W Scientifica system coupled with DAGE-MTI IR-1000 CCD. Scale bar: 20 μm. (**B**) The results showed more profound Ca^2+^ entry into the astrocyte differentiated through co-silencing of HSP27 and S100A16, than in the control cells. (**C**) There was a 17.9-fold enhancement of Ca^2+^ influx in astrocytes compared to control cells. Results calculated from three independent experiments. **: *p* < 0.01. (**D**) Ionic currents recorded in induced astrocyte (co-silencing of HSP27 and S100A16) by ramp protocol. Induced astrocytes exhibit outwardly K^+^ currents upon voltage stimulation (left panel). The rectifying profile was significantly reduced after addition of 10 mM of Tetraethylammonium, a potassium channel blocker (middle panel). Cells with luciferase (shLuc) silencing, used as control, showed a low and linear membrane conductance (right panel). (**E**) Representative traces of whole-cell voltage-clamp K^+^ currents recorded in induced astrocytes. Cells were subjected to 20 mV step depolarizations from − 100 mV to + 120 mV at a − 60 mV holding potential. Induced astrocyte showing an evoked rectifying profile upon voltage stimulation (left panel). Under 10 mM TEA addition, the profiles were significantly reduced (middle panel). Cells with luciferase silencing (shLuc) showed nearly no stimulation profile (left panel). (**F**) Decreasing percentage of ramp current at 100 mV after 10 mM TEA addition (*n* = 5 for shLuc, *n* = 6 for induced astrocyte. ***: *p* = 0.0003). (**G**) Resting membrane potential of shLuc cells and induced astrocytes. The resting membrane potential was recorded before membrane activation (*n* = 6 for shLuc, *n *= 8 for induced astrocyte. **: *p* = 0.0023)

We also investigated the function of the astrocytes derived from co-silencing of S100A16 and HSP27 in PDMCs. For functional experiments, astrocytes were induced by co-silencing of shHSP27 and S100A16 in PDMCs for 18 days (D18), while cells silenced with shLuc were used as control. First, we assessed their capacity to generate intracellular calcium waves after thrombin application. This well-established method to characterize the function of astrocytes involves a protease-activated receptor (PAR) expressed in functional astrocytes, which is activated by thrombin and enables Ca^2+^ entry into cells [20]. After differentiating PDMCs into astrocytes by HSP27 and S100A16 co-silencing, we added thrombin to activate PAR and examined Ca^2+^ entry by Calbryte 520 calcium dye. The results showed that thrombin addition led to more robust Ca^2+^ mobilization in the astrocyte-like cells than in control cells (Fig. 5B). After three independent experiments, the Ca^2+^ entry showed a nearly 17.9-fold increase on average in the differentiated astrocytes compared with control cells (Fig. 5C).

We next examined the electrophysiological properties of the induced astrocytes by patch-clamp recordings. First by ramping (Fig. 5D) and then by voltage-step (Fig. 5E) to investigate their ability to generate potassium currents. With respect to the former, induced astrocytes showed a large outwardly rectifying potassium conductance (Fig. 5D, left panel). On the contrary, control cells showed a linear conductance (Fig. 5D, right panel). By adding 10 mM tetraethylammonium (TEA), a potassium channel blocker, into the buffer, the induced astrocyte displayed a reduced outwardly rectifying potassium conductance (Fig. 5D, middle panel) indicating the existence of potassium channels. Finally, when the voltage-step protocol was applied to the induced astrocytes, we found typical outward potassium currents with rapid activation kinetics, partial slow inactivation, and a response magnitude proportional to the applied voltage (Fig. 5E, left panel). In control cells, nearly no responsive activation phase was evoked (Fig. 5E, right panel). Also, by adding 10 mM TEA, the response magnitude was drastically reduced (Fig. 5E, middle panel) indicating the existence of potassium channels in the induced astrocytes. At 100 mV induced astrocyte exhibited a nearly 66% decrease in response magnitude while control cells showed only an 8% decrease in average (Fig. 5F). We also recorded their resting membrane potential (Fig. 5G). The more negative resting membrane potential of induced astrocytes than control cells was in agreement with the properties of astrocytes [26]. The results of functional characterization showed that the cells derived from co-silencing of HSP27 and S100A16 in PDMC were able to respond to external stimuli and behave as astrocytes.

## Discussion

We previously demonstrated that HSP27 downregulation enhances PDMC differentiation into glutamatergic neurons under chemical induction [4]. Herein, we describe how co-silencing of HSP27 and S100A16 directs PDMCs to spontaneously differentiate into astrocytes without chemical induction. The main role of HSP27, regarded as a differentiation pathway keeper, is to prevent neural differentiation by interacting with procaspase 3, which needs to be activated at the beginning of differentiation. Therefore, the downregulated HSP27 expression level is beneficial for neural differentiation. Currently, little is known about S100A16; and almost all publications focus on its association with cancer. S100A16, a calcium-binding protein belonging to the S100 superfamily, is mostly involved in tumor progression [27]. It forms a homodimer, with two Ca^2+^ ions interacting in the EF hand of each subunit [28]. To date, there is no other report depicting the role of S100A16 in astrocyte differentiation. This is the first report on S100A16’s involvement in neural differentiation.

The identification of crucial molecules and the mechanisms controlling neural differentiation is a major step toward the successful treatment of neurodegenerative diseases. One of the major age-related neural degenerative diseases, AD accounts for the largest proportion (65%–70%) of dementia cases in the aged population [29, 30]. In this study, we used a double-cross-screening strategy with omics data derived from our stem cell differentiation model and an AD database to successfully identify a crucial combination of downregulated genes involved in astrocyte generation. The main function of astrocytes in the CNS is to maintain homeostasis in processes such as neurotransmitter uptake and recycling, synaptic activity modulation and ionic balance [31]. Many studies have shown that astrocyte function is altered in brains of patients with neurodegeneration [32]. For example, the presence of amyloid beta abnormally regulates gliotransmission and neurotransmitter uptake and alters calcium signaling in astrocytes [33]. The astrogliosis in AD is thought to be responsible for changes in critical molecule expression and morphology in astrocytes [34], changes that result in scar formation and inhibition of axon regeneration [35]. Through our screening strategy, we successfully found S100A16 in the AD dataset which was downregulated in the neuron-neogenesis dataset.

The double-cross-comparison strategy used here was proven to be an effective approach for the identification of molecules crucial for neural differentiation. The genes revealed through this strategy were sufficient to induce PDMCs to differentiate into cells that possess the functional and morphological characteristics of astrocytes. In conclusion, our findings provide molecular insights as well as a good astrocyte differentiation cell model for potential future application in human regenerative medicine.

## Supplementary Information

Below is the link to the electronic supplementary material.

Supplementary file1 (DOCX 30 KB)

Supplementary file2 (DOCX 364 KB)

## Data Availability

The data that support the findings of this study are available on request from the corresponding author.
