# Supplementary material for: Downregulated Calcium-Binding Protein S100A16 and HSP27 in Placenta-Derived Multipotent Cells Induce Functional Astrocyte Differentiation
Source: Stem Cell Rev Rep. 2022 Jan 21;18(2):839–52. doi: 10.1007/s12015-021-10319-3 (PMC8930865; doi:10.1007/s12015-021-10319-3)
Supplement: Supplementary file 1 — Supplementary file1 (DOCX 30 KB) [file 12015_2021_10319_MOESM1_ESM.docx]

**Supplements**

**Materials and methods**

**Shotgun proteomics-Protein digestion, dimethyl labeling, and strong cation exchange**

Total cellular lysates were acetone-precipitated and resuspended in a denaturing buffer containing 50 mM triethylammonium bicarbonate (TEABC) and 8 M urea. The protein concentration was determined with a BCA Protein Assay Kit (Thermo Scientific Pierce, Rockford, IL, USA) and adjusted to 1 mg/mL using denaturing buffer. The protein extracts were reduced with 10 mM dithiothreitol at room temperature for 30 min and alkylated with 50 mM iodoacetamide at room temperature in the dark for 30 min. The alkylated protein extracts were digested with Lysyl Endopeptidase (WAKO, Tokyo, Japan) (100:1 protein:trypsin ratio, w/w) at room temperature for 3 hr. After dilution with a 4-fold volume of 50 mM TEABC, the protein extracts were further digested with Sequencing-Grade Modified Trypsin (Promega, Madison, WI, USA) in a 100:1 protein:trypsin ratio (w/w) at room temperature overnight. The resulting tryptic peptides were desalted with reversed-phase StageTips, as previously described (Rappsilber, Mann, & Ishihama, 2007).

Dimethyl labeling of the tryptic peptides was performed as previously described (Boersema, Raijmakers, Lemeer, Mohammed, & Heck, 2009). The desalted peptides were vacuum-dried and resuspended in 100 mM TEABC. The peptide concentrations were estimated with a BCA Protein Assay Kit and adjusted to 0.25 mg/mL using 100 mM TEABC. For the dimethyl labeling of peptides, 100 μL of each peptide sample was mixed with 4 μL of 4% (v/v) formaldehyde solution and 4 μL of 0.6 M sodium cyanoborohydride. In this study, the tryptic peptides from IBMX-treated and control cells at each time point were differentially dimethyl-labeled with isotope-labeled (heavy) formaldehyde (^13^CD_2_O) and non-isotope-labeled (light) formaldehyde, respectively. After incubation at room temperature for 1 h, the labeling reactions were stopped by adding 16 μL of 1% (v/v) ammonia solution. Equal amounts of the heavy-labeled and light-labeled peptides were mixed and desalted as previously described.

The combined dimethyl-labeled peptides were further separated by SCX StageTips (Rappsilber et al., 2007). To elute the peptides, 20-500 mM ammonium acetate with 15% acetonitrile (ACN) was employed, resulting in 5 fractions. All eluted fractions, including the flow-through fraction, were desalted, vacuum-dried, resuspended in a sample loading buffer containing 2% ACN and 0.5% trifluoroacetic acid (TFA), and subjected to downstream nanoLC-MS/MS analyses.

**Shotgun proteomics-*NanoLC-MS/MS analyses***

Each dimethyl-labeled SCX fraction was analyzed by nanoLC-MS/MS analysis using an LTQ-Orbitrap XL mass spectrometer (Thermo Fisher Scientific, Bremen, Germany) equipped with a Dionex Ultimate 3000 RSLCnano System (Germering, Germany). The peptides resuspended in the sample loading buffer were directly loaded onto an in-house-pulled 15-cm-tip column (100-µm i.d.) packed with 3-µm ReproSil-Pur 120 C18-AQ reversed-phase beads (Dr. Maisch HPLC GmbH, Ammerbuch-Entringen, Germany) (Ishihama, Rappsilber, Andersen, & Mann, 2002). The injection volume was 5 μL, and the flow rate was 500 nL/min. The mobile phases were 0.5% acetic acid in water (Buffer A) and a mixture of 0.5% acetic acid and 80% ACN (Buffer B). The peptides were eluted with a gradient of 5% to 10% Buffer B in 5 min, 10% to 40% Buffer B in 120 min, 40% to 99% Buffer B in 5 min, and 99% Buffer B for 10 min.

The LTQ Orbitrap XL was operated in data-dependent mode, switching automatically between a full MS scan and MS/MS acquisition. A spray voltage of 2400 V was applied. The full MS scan spectra (*m/z* 300–1600) were acquired with a resolution of 60,000 at *m/z* 400 in the Orbitrap analyzer after a target value of 5 x 10^5^ was reached. The top 10 most intense precursor ions with charge states ≥ +2 were sequentially isolated with an isolation width of 2 Da and a target value of 1 x 10^4^. The selected precursor ions were fragmented using collision-induced dissociation in the linear ion trap with a 35% normalized collision energy. The activation q was set to 0.25, and the activation time was set to 30 ms. The dynamic exclusion time was 90 s, with an exclusion list size of 500. A lock mass of *m/z* 445.120024 was applied in all LC-MS/MS analyses (Olsen et al., 2005). In this study, all SCX fractions were analyzed three times.

**Shotgun proteomics-*MS data processing and analyses***

The raw files from LC-MS/MS were analyzed together using MaxQuant software, version 1.3.0.5 (Cox & Mann, 2008). The peak lists were derived from the MS/MS data with the 6 most abundant peaks per 100 Th. Using the Andromeda search engine in MaxQuant (Cox et al., 2011), the derived peak lists were searched against a human protein database with 20,226 sequence entries (Swiss-Prot, version 2012_11). The parameters for the database search were a precursor mass tolerance of 6 ppm, a fragment ion mass tolerance of 0.5 Da, trypsin specificity allowing for up to 2 missed cleavages, cysteine carbamidomethylation as the fixed modification, N-acetylation of proteins and oxidation of methionine as variable modifications, and a minimum peptide length of 7 amino acids. The Andromeda search results were further processed using MaxQuant. To achieve reliable identifications, the false discovery rates (FDRs) at the peptide and protein levels were set to 0.01.

Quantitation of the proteins identified in Andromeda was performed using MaxQuant based on quantification of the peak areas of the dimethyl-labeled peptide pairs (mass difference: 6 Da). To maximize the number of quantifiable peptides, re-quantification and matching between different runs with a 2-min LC time window were enabled in MaxQuant software. All raw data was uploaded to jPOSTrepo, an international standard data repository for proteomes, with the ID of JPST000694.

**Immunoblotting**

Cell lysates with the same amounts of proteins were subjected to 12% SDS–PAGE. After electrophoresis, the proteins in the gels were transblotted onto polyvinylidene fluoride membranes (25 mM Tris [pH 8.3], 192 mM glycine, 0.1% SDS, and 15% methanol) using a Semidry Transphor unit (Nihon Eido, Tokyo, Japan) at a constant current of 60 mA for 1 h. After that, 5% defatted milk in TBST buffer (20 mM Tris [pH 7.4], 150 mM NaCl, and 0.05% Tween 20) was used to blocked the membranes for 1 h. Then, the membranes were probed with primary antibodies in TBST buffer for 1 h at room temperature. The primary antibodies and dilutions used were as follows: anti-ALDH1A1 (1:500, #ab89431, Abcam, MA, USA), anti-FKBP7 (1:500, #ab69350, Abcam), anti-PDGFRA (1:250, #GTX63209, GeneTex), anti-COTL1 (1:500, #ab93427, Abcam), anti-TPM3 (1:200, #GTX113568, GeneTex), anti-S100A16 (1:200, ab130419, Abcam), anti-PLCB3 (1:500, #ab52199, Abcam), anti-ENAH (1:500, #ab92849, Abcam), anti-ANKRD13A (1:500, #GTX107299, GeneTex), anti-HSP27 (1:1,000, #ab1426, Abcam), anti-CCRN4L (1:500, #ab22850, Abcam), anti-CASP3 (1:200, #9662, Cell Signaling), anti-MT1E, (1:250, #NBP1-87740, NOVUS), anti-CKAP4 antibody (1:200, #AF7355, R&D Systems), anti-S100A16 antibody (1:500, #AF5180, R&D Systems), and anti-GAPDH (1:200, #AM4300, Ambion). The membranes were then incubated with appropriate horseradish peroxidase-conjugated anti-rabbit or anti-mouse antibodies (1:5,000, #A0545 or #A4416; MilliporeSigma, St. Louis, MO, USA). The HRP signals were detected upon addition of an HRP substrate (Western Lightning Chemiluminescence Reagent Plus, PerkinElmer, MA, USA) and visualized with a FluorChem FC2 Gel Imaging System (Alpha Innotech/Bio-Techne, CA, USA). The Image Studio Lite software were applied to digitalize the band intensity. (Ver5.2, LI-COR Biosciences, NE, USA).

**Total RNA extraction and quantitative real-time PCR.**

An RNeasy Mini Kit (Qiagen, Hilden, Germany) was used to extract total cellular RNA, and cDNA was synthesized from 2 μg of each RNA sample using a SuperScript III First-Strand Synthesis System (Invitrogen, Carlsbad, CA, USA). Before qRT–PCR, each RNA sample was quantified using a NanoDrop ND-1000 spectrophotometer (Thermo Fisher Scientific, Waltham, MA, USA) and qualified using a Bioanalyzer RNA Nano 6000 Chip Kit (Agilent Technologies, Santa Clara, CA, USA). The relative levels of the mRNAs of interest were quantified using qRT–PCR in the presence of a TaqMan probe and TaqMan Master Mix (Roche Diagnostics GmbH, Mannheim, Germany). The primers used for the amplification of each gene are listed in Table S1. For quantification, the expression levels of the genes of interest were normalized by the expression level of 18s rRNA. LightCycler Software (Version 4.05, Roche Diagnostics GmbH, Mannheim, Germany) was used to generate quantitative data.

**Immunofluorescence and Laser scanning confocal fluorescence images**

The cells were fixed and blocked, and then incubated with primary antibodies overnight at 4 °C. The primary antibodies used were as follows: anti-MAP2 (1:200, #AB5622; Chemicon/Merck Millipore, MA, USA), anti-Tubulin beta III isoform (Tuj1, #MAB1637, Chemicon/ Merck Millipore), anti-vGLUT1 (1:200, #AB72311, Abcam, Cambridge, UK), anti-GFAP (1:500, #MAB3402, Chemicon/ Merck Millipore), anti-ALDH1L1 (1:250, #ab56777, Abcam), and anti-GS (1:100, #SC-6640, Santa Cruz), anti-S100B (1:200, #ab52642 Abcam), anti-SOX9 (#ab185230, Abcam) and anti-KIR4.1 (1:250, #ab240876 Abcam). After that, the cells were incubated with appropriate fluorescein-labeled secondary antibodies for 1 h at room temperature, and then 4’,6’-diamidino-2-phenylindole (DAPI) was added to visualize the cell nuclei. Immunofluorescence images were captured using a Nikon Eclipse 80i fluorescence microscope (Nikon Instruments, Tokyo, Japan) and Laser scanning confocal fluorescence images were capture by Leica TCS SP8X (Leica Microsystems, Wetzlar, Germany).

Table S1A. Primers used in ROCHE qRT-PCR system

Table S1B. Primers designed by Qiagen.
