# Supplementary material for: Downregulated Calcium-Binding Protein S100A16 and HSP27 in Placenta-Derived Multipotent Cells Induce Functional Astrocyte Differentiation
Source: Stem Cell Rev Rep. 2022 Jan 21;18(2):839–52. doi: 10.1007/s12015-021-10319-3 (PMC8930865; doi:10.1007/s12015-021-10319-3)
Supplement: Supplementary file 2 — Supplementary file2 (DOCX 364 KB) [file 12015_2021_10319_MOESM2_ESM.docx]

**Supplement S2**

**The effects of the combination of 4-gene silence (HSP27, S100A16, PLCB3, MT1E) and the combination of 3-gene silence in neural or astrocyte differentiation.**

Each combination of three-gene silencing as well as four-gene silencing were performed (Figure S2A). The results of twelve days after virus infection showed that neither one of them have better astrocyte differentiation ability than shHSP27 plus shS100A16. The differentiated astrocytes of each experimental groups were quantified (Figure S2B) and none of them over 10 percent compare to the results of shHSP27 plus shS100A16 manipulation which with the differentiation percentage around 15% twelve days after virus infection.


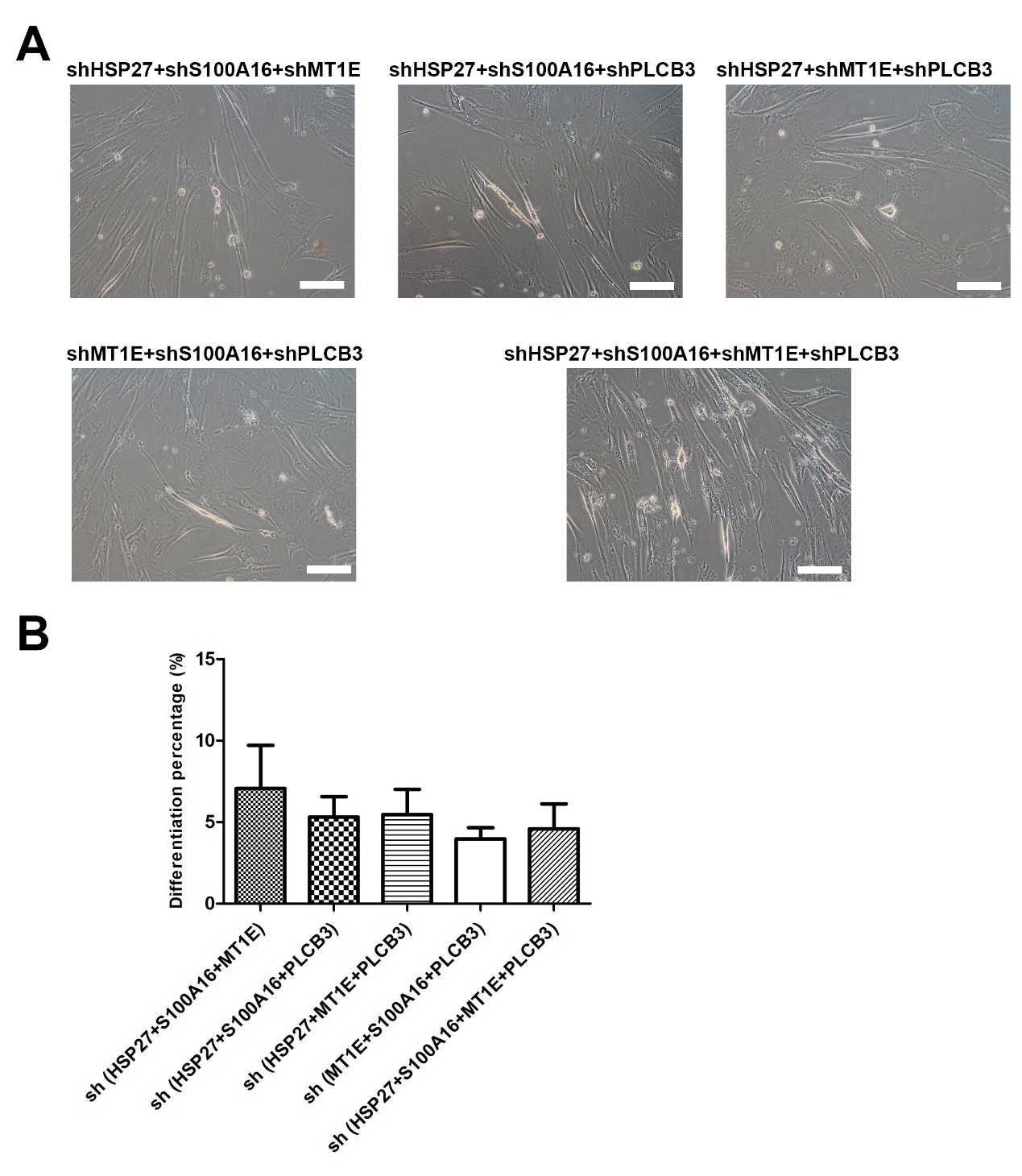


**Figure S2 (A) The cell morphology with combination of various gene silencing.** The cell were infected with various combinations of the virus contained shHSP27, shS100A16, shMT1E or shPLCB3. The cell images were taken 12 days after infection. Scale bar: 100 μm.

**(B) The astrocyte differentiation percentage of combination of various gene silencing.** The results were obtained from six randomly selected pictures of each group from three independent experiments and the differentiated astrocytes were count according to their morphology. The differentiation percentage were calculated using induced astrocyte number divided with cell number. Abbreviation: sh(HSP27+S100A16+MT1E) means the cells infected with virus containing short hairpin RNA specific against HSP27, S100A16 and MT1E; sh(HSP27+S100A16+PLCB3) means the cells infected with virus containing short hairpin RNA specific against HSP27, S100A16 and PLCB3; sh(HSP27+MT1E+PLCB3) means the cells infected with virus containing short hairpin RNA specific against HSP27, MT1E and PLCB3; sh(MT1E+S100A16+PLCB3)means the cells infected with virus containing short hairpin RNA specific against MT1E, S100A16 and PLCB3; sh(HSP27+S100A16+MT1E+PLCB3) means the cells infected with virus containing short hairpin RNA specific against HSP27, S100A16, MT1E and PLCB3.
